# Response to intervention for high school students: examining baseline word reading skills and reading comprehension outcomes

**DOI:** 10.1007/s11881-022-00253-5

**Published:** 2022-03-08

**Authors:** Michael Solis, Paulina Kulesz, Kelly Williams

**Affiliations:** 1 Graduate School of Education, University of California Riverside, 1207 Sproul Hall, Riverside, CA 92521 USA; 2 Texas Institute for Measurement Evaluation and Statistics, University of Houston, Houston, TX USA; 3 School of Education, Indiana University, Bloomington, IA USA

**Keywords:** Adolescent literacy, High school, Reading comprehension, Word reading

## Abstract

The purpose of this post hoc analysis was to analyze if pre-intervention word reading skills contributed to intervention response on reading comprehension outcomes. High school students with reading difficulties were randomized to a business as usual (BaU) or treatment condition that provided 2 years of an intensive, multicomponent word reading and reading comprehension intervention. Participants were assessed on measures of word reading and reading comprehension for pretest and reading comprehension only for posttest. Findings revealed no statistically significant differences with word-level fluency modeled as a continuous variable between treatment and control on reading comprehension. Regardless of assignment to condition, higher word-level fluency scores predicted higher posttest outcomes on years 1 and 2 reading comprehension scores.

By the time students enter high school, their teachers expect them to be proficient with word-level reading and reading comprehension skills necessary to acquire information from complex grade-level texts; however, according to the National Assessment of Education Progress (NAEP), 67% of students were at or below the basic level of reading proficiency by the end of 8th grade (Hussar et al., 2020). Results of reader profile studies of adolescents show that many students with the lowest levels of reading comprehension also demonstrate difficulties with accurate and fluent word reading and language comprehension (Brasseur-Hock et al., 2011; Cirino et al., 2013; Hock et al., 2009).

Hock et al. (2009) examined the reading achievement of 345 students in grades 8 and 9, and found that struggling readers (i.e., who scored below the 40th percentile on the Kansas Reading Assessment) had significantly lower mean standard scores than proficient readers on measures of word reading accuracy, reading fluency, vocabulary, and language comprehension (Hock et al., 2009). Brasseur-Hock et al. (2011) extended these findings with 319 participants with complete outcome data from Hock et al.’s (2009) study. Fifty-one percent of the participants with comprehension difficulties (i.e., struggling comprehenders and low average comprehenders) had moderate or severe global weaknesses, which meant they scored at least one standard deviation below the mean on all measures of reading achievement (Brasseur-Hock et al., 2011).

Cirino et al. (2013) measured reading outcomes (i.e., word reading accuracy, word and text reading fluency, comprehension, and combined fluency and comprehension) in a sample of 1,785 typical and struggling readers (i.e., those that scored below a predetermined cut score on the state reading assessment) in grades 6 through 8. Among the 846 struggling readers, 49% scored below the 25th percentile on three or more of these measures, and 47% scored below the 25th percentile on word reading accuracy (Cirino et al., 2013). Brasseur-Hock et al. (2011) reported approximately 70% of struggling comprehenders (i.e., those with the lowest reading comprehension) had moderate or severe global weaknesses in all areas assessed compared to only 19% of the low average comprehenders. Similarly, students with learning disabilities (LD) scored significantly lower than their peers without disabilities on all outcome measures, with the largest differences being detected for word reading accuracy outcomes between the two groups (22 pt. standard score difference) (Hock et al., 2009). The findings from these studies demonstrate that adolescents with the most significant reading comprehension difficulties (including students with LD) also frequently have below average word-level reading scores (Brasseur-Hock et al., 2011; Cirino et al., 2013; Hock et al., 2009). Much less is known about the relation of these components for high school students than for any other group of at-risk readers (Wang et al., 2019).

## Theoretical and empirical framework

In the Simple View of Reading (SVR), reading comprehension is conceptualized as the product of two component skills, decoding and linguistic comprehension (Gough & Tunmer, 1986; Hoover & Gough, 1990). Decoding, a word-level skill, involves rapidly and efficiently retrieving words from memory, and linguistic comprehension consists of the literal and inferential construction and interpretation of the meaning of those words (Hoover & Gough, 1990; Hoover & Tunmer, 2018). To extend the understanding of the SVR, the relations between word reading, language, and comprehension have been investigated through confirmatory factor analysis and latent variables as part of structural equation models to predict reading comprehension across elementary and high school grades (Foorman et al., 2018, 2020). In both studies by Foorman and colleagues, the findings for older students (high school) showed less stability and less common variance across decoding and comprehension. In consideration of the SVR, the multiplicative association between decoding (word reading) and reading comprehension diminished for older students. Foorman and colleagues posited consideration of an additive model for the upper grades (Foorman et al., 2018, 2020).

Other studies have also shown marked differences regarding relations between word reading and comprehension for younger readers when compared to older readers (García and Cain, 2014; Wang et al., 2019) The meta-analysis conducted by Garcia and Cain (2014) estimated a correlation between decoding and comprehension of 0.80 for younger readers (age < 10 years) and 0.47 for older readers (age > 11 years). Similarly, the correlation between decoding and comprehension reported by Wang et al. (2019) was 0.55 for older students (5th grade and above). However, when Wang and colleagues (2019) also took into account the decoding ability in addition to the grade placement, the correlations for students with lower decoding ability were much smaller (grades 5–12). For example, the analysis for 9th graders reported an overall correlation between decoding and reading comprehension of 0.54. However, for 9th graders with low decoding levels, the findings diverged drastically with correlations of 0.04 being estimated. Wang et al. (2019) proposed the decoding threshold hypothesis (DTH) which asserts that for performance below a certain threshold, the association between word reading and comprehension is no longer discernable. This implies that word reading ability may need to be above a certain threshold in order for meaningful gains to be possible for comprehension.

## Intervention research for adolescents

Adolescents with significant reading disabilities including those with dyslexia require intensive reading interventions that address multiple components of reading in order to improve their reading achievement (Vaughn & Fletcher, 2012; Vaughn et al., 2019). Results from several recent systematic reviews and meta-analyses demonstrate that intensive, multicomponent reading interventions lead to improved reading outcomes for adolescents with reading difficulties and disabilities (Edmonds et al., 2009; Herrera et al., 2016; Scammacca et al., 2016; Wanzek et al., 2013). Across these systematic reviews, a total 141 experimental and quasi-experimental studies were included. Of those studies, only 23 utilized participants’ samples that exclusively had students above 8th grade and only one experimental study with components of word reading and comprehension reported statistically significant results favoring the treatment (Lang et al., 2009).

### Multicomponent reading intervention research for high school students

Despite an increase in the number of studies published over the last 15 years, there are still very few multicomponent studies that address word-level reading and reading comprehension for high school students (Lang et al., 2009; Solis et al., 2018; Vaughn et al., 2015). In a large-scale study (*N* = 1,265), Lang et al. (2009) compared three multicomponent reading interventions provided for 90 min per day for 1 year to 9th graders. Intervention conditions included READ 180® (Hasselbring et al., 2020), the REACH System 2002 (REACH; https://www.mheducation.com/prek-12/program/reach-system-2002), and Reading Instruction through Strategy Enhancement (RISE; Lefsky, 2004) to a comparison condition. All of these commercialized intervention packages provide a combination of word-level instruction and reading comprehension instruction of connected text. For a subset of students with most severe reading problems, there were no statistically significant differences between the intervention conditions and the comparison condition on the criterion-referenced state reading exam.

High school reading interventions have also been designed to focus on word-level fluency and comprehension instruction that emphasizes expository text to support content area (social studies and science) learning expectations. In a small-scale experimental study of 9th graders (*N* = 91) with reading difficulties, Solis et al. (2018) provided an intensive 1-year intervention with units of comprehension instruction aligned with social studies and science topics (i.e., human geography) coupled with high interest chapter book units. Instruction also included daily word-level fluency practice by reading discrete skill phonetic and irregular word lists with the purpose of building speed, accuracy, and expression. Findings from this study were mixed. Repeated measures of ANCOVAs indicated statistically significant effects favoring the treatment for a proximal vocabulary measure (*n*_p_^2^ = 0.19) and one standardized measure of reading comprehension (*n*_p_^2^ = 0.02). Statistically significant effects were not detected with a second standardized measure of reading comprehension. The data analysis in this study did not take into account pre-wording reading skills in the analysis.

In a multi-year longitudinal study investigating reading interventions and dropout prevention for high school students, Vaughn et al. (2015) compared two intervention conditions (reading intervention only, reading intervention plus dropout prevention) to a business as usual (BAU) comparison condition. High school students (*N* = 375) with reading problems participated in the 2-year study during their ninth and tenth grade years. This study employed a multicomponent intervention with word-level instruction and comprehension instruction associated with content area learning topics organized by units of study. Over the duration of the intervention, instruction shifted from emphasizing more word-level instruction initially to reading comprehension instruction emphasizing application of skills. Findings over the 2-year study indicated students in the reading intervention condition demonstrated significant gains on reading comprehension outcomes (*ES* = 0.43) compared to students in the BAU condition. Interestingly, all of these multicomponent reading intervention studies conducted with high school students included components of word reading, yet word reading scores were not included in the analysis.

## Intervention studies of baseline word reading skills and comprehension

Findings from intervention studies that include analysis of how word reading may be associated with reading comprehension across treatment and BAU conditions provide further insight into these relations for students with reading problems. By including well-defined intervention treatments and comparison conditions, knowledge of the instructional components further contextualizes the findings from reader profile studies and empirical studies of the SVR. Since considerably less is known about how high school students’ response to intervention might differ based on their pre-intervention word-level reading skills, we review studies utilizing samples of middle school and high school students.

In a study of middle school students, Clemens et al. (2019) conducted a secondary analysis of pre-word reading skills as part of an intervention study. The data analysis from the main effects study (Fogarty et al., 2017) reported statistically significant effects favoring the treatment for reading comprehension (*ES* = 0.14), proximal vocabulary (*ES* = 0.43), and silent reading efficiency (*ES* = 0.28). The intervention consisted of a circuit-based approach with components of word reading, fluency, vocabulary, and comprehension. Clemens et al. (2019) conducted a secondary analysis to investigate the extent to which pretest word reading, reading fluency, and vocabulary moderated the effects of reading comprehension outcomes for students with reading difficulties in grades 6–8. Results indicated that word reading did not moderate the effects of reading comprehension; rather, reading fluency moderated the effects of reading comprehension for students with lower scores compared to students with higher scores in the intervention, and students in the comparison condition. These findings diverge from the influence of pre-word reading skills detected in the studies of upper elementary students. However, one common characteristic of all the studies from upper elementary and this middle study is the intervention treatments that include components of word reading and comprehension.

In a small underpowered study, Solis et al. (2015) examined the effects of a reading comprehension only intervention on the reading outcomes of 44 ninth grade students with reading comprehension difficulties with word reading scores in the low average range. Participants were randomized to treatment (*n* = 25) and comparison (*n* = 18) conditions prior to being assessed at pretest on word reading and pre and post on measures of silent reading efficiency and comprehension outcomes. The main effects model showed no statistically significant differences between the treatment and comparison conditions on any measures of reading comprehension. However, a follow-up analysis that categorized students as low decoders (TOWRE standard score < 93) and high decoders (TOWRE standard score > 93) indicated a statistically significant interaction between decoding ability and comprehension, such that participants with higher word-level reading skills performed significantly better on passage comprehension and inferencing than participants who were low decoders (TOWRE standard scores < 93) (Solis et al., 2015). While this finding is potentially meaningful, the authors cautioned over generalizing it based on the small sample size and the analysis being underpowered, which increases the confidence intervals and reduces the external validity.

## Purpose of the study

Within the current literature, it is evident that more studies are necessary to strengthen the empirical base of knowledge on how initial word reading skills may impact outcomes within the framework of interventions being provided, especially for adolescents with reading difficulties and disabilities. Based on findings from reader profile studies of adolescents and the mixed findings from the small body of intervention studies investigating word-level variables, this study may provide insights to understanding how to design and deliver interventions for adolescents with significant reading difficulties and disabilities. Therefore, in the current study, we explored relations between pre-intervention word reading performance and posttest reading comprehension after year 1 and year 2 of an intensive reading intervention for high school students with low performance on word reading and comprehension.

We conducted a secondary data analysis using data from Vaughn et al. (2015) to investigate whether pre-intervention word-level reading skills contributed to response to intervention with reading comprehension outcomes for high school students with significant reading difficulties. This study extends the findings conducted with middle school students to better understand the relations of word reading and comprehension as part of outcomes of intervention studies with comparison conditions. To date, no studies have reported on baseline word reading ability on response to intervention for high school students receiving a multicomponent intervention of word reading and comprehension (Daniel et al., 2021). We aimed to compare the differential impact of word reading ability modeled as a continuous variable across treatment and comparison conditions on reading comprehension outcomes for ninth and tenth grade students with reading difficulties. Specifically, the purpose of the current study involving students with a variable range of reading difficulties was to answer the following research questions:

1. How do pretest word-level fluency scores affect response to intervention compared to BAU on year 1 and year 2 posttest reading comprehension outcomes?
2. How do pretest word-level fluency scores affect response to intervention compared to BAU in year 2 reading comprehension outcomes over and above gains in year 1 reading comprehension outcomes?

## Method

In accordance with the institutional review board (IRB) requirements and data agreements with the cooperating districts, we obtained the original deidentified data files (Vaughn et al., 2015). Data files included the pretest and posttest scores for students in the treatment and comparison conditions. A methodologist independent of the researchers involved with the development and implementation of the intervention conducted the secondary data analysis.

## Research design

Data for this study are taken from a 2-year randomized control trial comparing students receiving a multicomponent reading intervention (with or without dropout prevention) to students in a BAU condition (Vaughn et al., 2015). The intervention was provided for 2 years (9th and 10th grade). For purposes of this analysis, all students who received reading intervention were combined and compared to the BAU condition.

### Participants

#### Reading intervention teachers

The reading intervention teachers were hired, trained, and supervised by the research team. All interventionists had teacher licensure and experience working with students with reading difficulties. One week of professional development was provided prior to the intervention start date. Topics covered included the research design, features of effective instruction, and information regarding fidelity of implementation. This was followed by regular classroom instructional coaching and quarterly half-day booster sessions. One half-day session was provided prior to the beginning of year 2.

#### Students

Students from five middle schools that feed into three participating high schools in one urban district were screened for participation during their 8th grade year. The high schools were located in a large and diverse urban area in the southwestern USA. Across the high schools, the student makeup included 43.11% Hispanic, 25.51% White, 19.44% African American, 7.85% Asian, and 4.06% Native American or biracial. The percent of students classified as economically disadvantaged was 42.6%. See Table 1 for a breakdown of the student demographic data for this analysis.

**Table 1:** Student demographics

	Overall		Treatment		Comparison	
	*N*	*%*	*n*	*%*	*n*	*%*
Gender						
Male	209	61.47	94	27.65	115	33.82
Female	131	38.53	58	17.06	73	21.47
Race						
White	60	17.65	27	7.94	33	9.71
African American	111	32.65	52	15.29	59	17.36
Hispanic	150	44.11	67	19.71	83	24.40
Asian	19	5.59	6	1.76	13	3.83
English as a second language						
Yes	65	19.17	27	7.96	38	11.21
No	247	80.83	125	36.88	149	43.95
Special needs						
Yes	59	17.40	30	8.85	29	8.55
No	280	82.60	122	35.99	158	46.61

Note. *N* = 340 (a sample size used in path analyses). Please note there is one missing observation for English as a second language and special needs categories

### Selection criteria

Students were eligible for participation if their scores fell below passing or within the standard error of measure (cut score < 2200) on their 7th grade state reading accountability reading test (Texas Assessment of Knowledge and Skills [TAKS]; Texas Education Agency [TEA], 2004). At this point in the study, a total of 457 students were randomized within school across the treatments and BAU conditions. By the beginning of the following school year, the sample included 375 students. The attrition of the sample was as a result of several factors including student mobility, requested schedule changes, and student/parent refusal to participate. To determine if there was differential attrition across groups, a two-way analysis of variance was conducted on the baseline outcome variables (i.e., reading comprehension) by taking into account the treatment status, completer status, and any possible interactions (Cook et al., 1979). The findings revealed no statistically significant main effects for completer status, and no statistically significant interaction between condition status and completer status. The findings from the attrition analysis conducted of the data indicated that it was unlikely attrition influenced the observed effects of the intervention.

### Secondary data analysis sampling procedure

As part of the main effects study (Vaughn et al., 2015), there were two treatment conditions: treatment I students received the reading intervention, and treatment II students received the reading intervention and a dropout prevention intervention. For purposes of this analysis, we included all students who received the reading intervention as one treatment group, regardless of whether they received the dropout prevention intervention. From the deidentified dataset, we selected all students that were administered the outcome variables of interest. Participants were included in the analysis if scores were reported for the Test of Word Reading Efficiency-2 (TOWRE-2; Torgesen et al., 2012) and the comprehension subtest of the Gates MacGinitie Reading Test (MacGinitie et al., 2000). This further reduced the sample size (*N* = 340) though the attrition analysis contrasting samples equal to 375 and 340 did not yield statistically significant differences on the outcome measures. Sample sizes for pre and posttest measures are reported in Table 2.

**Table 2:** Standard scores and standard deviations for reading comprehension outcomes by treatment, pretest/posttest, and intervention year

		Pretest year 1			Posttest year 1			Pretest year 2			Posttest year 2		
		*M*	*SD*	*n*	*M*	*SD*	*n*	*M*	*SD*	*n*	*M*	*SD*	*n*
Comprehension SS	BAU	92.23	10.76	186	88.72	11.78	187	88.17	9.59	177	91.42	9.14	163
	Treatment	88.64	12.06	152	89.45	11.87	151	87.53	9.62	141	92.55	7.73	137
Word-level fluency composite SS	BAU	92.92	11.73	188	-	-	-	-	-	-	-	-	-
	Treatment	91.52	11.81	152	-	-	-	-	-	-	-	-	-

Note. *SS*, standard score; *BAU*, business as usual group; *N* = 340 (an actual sample size used in analyses). Listed in the table sample sizes (by treatment and intervention year) do not fully reflect the dropout study pattern (REF) because the current study only includes students with complete TOWRE-2 data. “-” = although word-level fluency was administered in years 1 and 2 of the dropout study, the current project only makes use of year 1 pretest score

### Measures

#### The Gates MacGinitie Reading Test (MacGinitie et al., 2000)

The comprehension subtest of the Gates MacGinitie Reading Test (GM-RT) was administered. The GM-RT is a group-administered, norm-referenced reading test that provides students with expository and narrative readings followed by multiple choice questions. Internal consistency reliability ranges from 0.91 to 0.93, and alternate forms reliability is reported as 0.80 to 0.87. Concurrent validity correlations for the GM-RT range from 0.72 to 0.87 (Morsy et al., 2010).

#### *Test of Word Reading Efficiency-2* (TOWRE-2; Torgesen et al., 2012)

The TOWRE-2 was administered during pretest only to determine word-level reading skills. The TOWRE-2 includes subtests of sight word reading and phonemic decoding efficiency. The alternate forms reliability coefficients were reported as 0.91 to 0.97 (Torgesen et al., 2012).

### Reading intervention

Intervention teachers provided 50-min classes 5 days per week for approximately 160 sessions per year for a total of about 320 sessions over 2 years. The instruction was delivered over two phases of instruction that focused on word study, vocabulary and comprehension of content area text topics, and engagement. The first phase during the first half of year 1 emphasized advanced word study strategy through the REWARDS Plus program (Archer et al., 2005). Students were taught how to identify orthographic patterns and understand the meaning of affixes, vowel-vowel combinations, corresponding sounds, and segmenting multisyllabic words into decodable chunks. Students learned six to eight vocabulary words that were selected from social studies and science textbooks on a weekly basis. Words were explicitly taught and reinforced daily through application activities of words and through exposure of the words within text. Students were taught comprehension strategies based on collaborative strategic reading (CSR) (Vaughn et al., 2011a, 2011b) using a six-step summary writing strategy (Brown & Day, 1983; Cordero-Ponce, 2000; Klingner et al., 2011). Interventionists provided explicit modeling and scaffolded support in applying the CSR strategies while reading social studies and science text associated with content area topics.

Phase II started during the second half of year 1. This phase continued to provide ongoing word-level instruction including the introduction of additional affix meanings. However, the emphasis of instruction shifted towards more practice with comprehension strategies being applied to instructional units taking place over six to eight classes with a primary focus on reading text associated with content area classes, with each unit including word study strategies of unfamiliar words and multisyllabic words. Vocabulary was explicitly introduced and reinforced daily (Vaughn Gross Center for Reading & Language Arts, 2010) and application of the six-step summary writing strategy continued to be applied to the use of the CSR strategies. Finally, each unit had an explicit discussion task designed to facilitate critical analysis and problem solving to support deeper understanding of the text. Student engagement was addressed by aligning the passages to content area course materials, providing specific content area learning goals, providing links to the relevance of content, and providing 10 min of student free choice for daily silent reading.

### Comparison condition

The reading intervention was provided as an elective course offering. Students in the business as usual (BaU) condition participated in other elective course offerings including fine arts, speech, computer skills, and technical applications. School-provided reading classes were not offered at any of the high schools.

### Implementation fidelity

Fidelity data were collected through by the research term by observing each teacher six times per year by a senior member of the research team familiar with the intervention. The fidelity form components focused on observed instruction with vocabulary, comprehension, discussion, motivation, and student engagement. Global indicators of classroom management and instruction were also coded. Twice per year reliability calibrations occurred by having the project director serve as a second observer and calculating interrater reliability of coding which was maintained at levels greater than 90%. The mean total fidelity score for year 1 was 84% and for year 2 90%.

### Data analysis

We computed two path analyses to address the research questions. In the analyses, year 1 and year 2 reading comprehension posttest scores were continuously distributed dependent variables. To address the first research question, year 1 and year 2 reading comprehension posttest scores were regressed on treatment (binary independent variable), interaction of treatment with year 1 word-level fluency pretest, year 1 word-level fluency pretest, and year 1 reading comprehension pretest with the latter two independent variables being continuously distributed and grand mean centered.

An analysis for the second research question was similar to the one for the first research question, except for regressing year 2 reading comprehension posttest score on year 1 reading comprehension posttest score in order to measure gains in year 2 over and above year 1. In both analyses, we tested an interaction hypothesis between treatment and word-level fluency skills in all possible ways. In other words, we considered interaction effects for both reading comprehension outcomes simultaneously, as well as one outcome but not the other. None of the interactions significantly predicted reading comprehension year 1 or year 2 posttest scores and were excluded from the final models. Similarly, year 2 pretest reading comprehension score did not yield statistically significant effects on the outcome, and was removed from the final models. Path analyses were estimated in Mplus 8.6 (Muthén & Muthén, 2017).

## Results

Standard scale scores and standard deviations for BAU and treatment groups by pretest and posttest as well as intervention year (year 1, year 2) for reading comprehension and word-level fluency are presented in Table 2. In examining possible effects of word-level fluency skills, we undertook a continuous rather than categorical approach. In other words, we looked at continuously distributed word-level fluency skills scores as opposed to scores based on cut-points. This perspective allowed us to preserve continuity of word-level fluency skills when examining individual differences in reading comprehension.

## Research question 1

There was no evidence of statistically significant interactions between the intervention assignment (BAU and treatment) and year 1 pretest word-level fluency scores on year 1 posttest reading comprehension ($\beta$ = − 0.07, *p* = 0.884) and year 2 posttest reading comprehension ($\beta$ = 0.29, *p* = 0.579), though an examination of regression coefficients for BAU and treatment students suggested differences in the magnitude of the relations between word-level fluency and reading comprehension in the two groups. Looking at the year 1 posttest reading comprehension, for BAU students, the relation of word-level fluency with reading comprehension was equal to 0.36 while for the treatment students, this relation was equal to 0.28. Looking at the year 2 posttest reading comprehension, for BAU students, the relation of word-level fluency with reading comprehension was equal to 0.25 while for the treatment students, this relation was equal to 0.54. As such, the relations were not uniform though the difference in the relations of word-level fluency with reading comprehension between the two groups did not yield statistically significant interactions. Higher word-level fluency was predictive of reading comprehension regardless of intervention assignment though the magnitude of relations between word-level fluency and reading comprehension differed depending on the group. Year 1 pretest reading comprehension was predictive of year 1 ($\beta$ = 0.51, *p* < 0.001) and year 2 ($\beta$ = 0.41, *p* < 0.001) posttest reading comprehension scores. Findings from the significance tests for the standardized regression coefficients of the path model for years 1 and 2 are presented in Table 3.

**Table 3:** Significance tests for standardized regression coefficients based on path model with years 1 and 2 posttest reading comprehension as outcomes

Year 1 posttest reading comprehension			
	$\beta$	*SE*	*p value*
Intervention (BAU and treatment)	0.168	0.502	0.737
Intervention × year 1 pretest word reading fluency interaction	− 0.073	0.499	0.884
Year 1 pretest word reading fluency	0.357	0.058	< .001
Year 1 pretest reading comprehension	0.508	0.047	< .001
Year 2 posttest reading comprehension			
	$\beta$	*SE*	*p value*
Intervention (BAU and treatment)	− 0.281	0.529	0.595
Intervention × year 1 pretest word reading fluency interaction	0.294	0.529	0.579
Year 1 pretest word reading fluency	0.252	0.069	< .001
Year 1 pretest reading comprehension	0.405	0.054	< .001

## Research question 2

The effect of treatment was statistically significant for year 1 reading comprehension posttest ($\beta$ = 0.14, *p* < 0.001), but not for year 2 reading comprehension posttest. The findings suggested that there was no booster when going from year 1 to year 2 intervention. The effects of word-level fluency skills on year 1 ($\beta$ = 0.08, *p* = 0.075) and year 2 ($\beta$ = 0.09, *p* = 0.081) posttest reading comprehension outcomes were marginal. Year 1 pretest reading comprehension was predictive of year 1 ($\beta$ = 0.66, *p* < 0.001) and year 2 ($\beta$ = 0.15, *p* = 0.029) posttest reading comprehension scores. Lastly, year 1 posttest reading comprehension was predictive of year 2 posttest reading comprehension ($\beta$ = 0.48, *p* < 0.001).

## Discussion

The secondary data analysis conducted for this study investigated whether pre-intervention word-level reading skills contributed to the response to intervention with reading comprehension outcomes for high school students with significant reading difficulties. The approach to analysis was to look at potentially differential impact of word reading ability modeled as a continuous variable across treatment and comparison conditions on reading comprehension outcomes for years 1 and 2 of the study. Findings revealed no statistically significant moderating effects of word-level fluency (modeled as a continuous variable) on a relation between intervention assignment and reading comprehension. However, despite not reaching statistical significance, the relations were not uniform across the conditions indicating difference levels of performance associated with assignment to condition. Those differences favor the treatment condition and align with the finding from the main effect study (Vaughn et al., 2015). However, when word reading was modeled as a continuous variable, regardless of assignment to a condition, higher word-level fluency scores predicted higher posttest outcomes on years 1 and 2 reading comprehension scores. In regards to research question 2, findings suggested statistical significance for year 1 reading comprehension but not for year 2 reading comprehension, indicating no additional increase in performance was detected from year 1 to year 2.

Investigating the relations of word reading, language, and reading comprehension within a framework of intervention provides an opportunity to further contextualize how these associations intersect by taking into account individual differences in performance due to baseline reading scores and assignment to condition. This study provides an opportunity to have an additional insight into how these constructs interact within the framework of well-defined intervention implemented with fidelity and a business as usual (BAU) condition across pre- and post-measures.

Participants in this study had pre-word-level standard scores that were approximately 0.5 standard deviations below the norming group average indicating near average word reading rather than very low word reading ability. This may suggest that the scores were not low enough to expect to find moderating effects regardless of the emphasis on word reading within the intervention or the assignment to condition. As posited by the decoding threshold hypothesis (Wang et al., 2019), students must be above a certain level of performance with decoding to expect growth in comprehension. In this study, this appears to be the case, regardless of the instruction received.

On the contrary, an intervention study of 9th graders with reading difficulties who received an intervention that only focused on comprehension (Solis et al., 2015) reported no statistically significant main effects, yet found a statistically significant interaction between treatment condition and decoding ability on two of three standardized measures of reading comprehension. These findings indicated the intervention to be differentially beneficial to students with higher decoding skills. Findings from the study supported the idea that students with lower word reading skills will continue to need instruction with word study component of the intervention. Since no instructional time was devoted to word reading, it may mean that students with higher decoding benefitted more from the intervention more closely aligned to their instructional need. However, it is important to note the limitations of these findings based on the initial underpowered sample size (*N* = 41) with even smaller group sizes of low decoders and high decoders across treatment and BAU for the post hoc analysis (*range* 8–13).

In the secondary data analysis of an intervention study investigating the associations of word reading and comprehension for middle school students, Clemens et al. (2019) reported that pre-word reading skills did not moderate outcomes of reading comprehension; however, effects of pre-reading fluency levels of connected text were detected showing that students in the intervention condition who had lower baseline levels of fluency had larger gains on comprehension than students with higher pretest fluency scores and for students in the comparison condition. One possible explanation is that reading fluency functions as a proxy of reading comprehension (Fuchs et al., 2001; Reschley et al., 2009), whereas semantic and syntactic knowledge contribute to the reciprocal relation between fluency and comprehension (Klauda & Guthrie, 2008). More familiarity with the structure of words and their meaning may ease the processing demands and facilitate automaticity of reading connected text (Perfetti & Stafura, 2014). For the few studies of high school students that yielded significant effects for comprehension, the interventions included both word reading (i.e., decoding) and reading comprehension (Lang et al., 2009; Lovett et al., 2012; Solis et al., 2018; Vaughn et al., 2015). While speculative, this may indicate that this combination of instruction is supporting automaticity with reading—automaticity that is not necessarily being detected due to the limitations with measurement of these separate yet related constructs.

### Limitations

The findings from this study are limited by the small number of observed variables representing the constructs of word reading and comprehension. Multiple measures of these constructs would provide the opportunity for latent variables approach, and therefore reduce the error that is inherent with measurement. However, both measures have strong psychometric properties. The findings are also limited by the size and makeup of the sample. The sample size in this study was not adequately large enough to conduct follow-up analysis on a subgroup of participants with the lowest scores on word reading measures (i.e., two standard deviations below the norming average). In other words, the current study was underpowered to conduct analyses exclusively involving participants with the most severe word reading difficulties through our approach to model word reading as a continuous variable as methodological rigor to understanding the performance of participants across different ability levels. While there was diversity with the ethnic and racial makeup, very few students were classified as English learners which are growing population of students. It is unclear if findings would generalize to students whose first language is not English.

### Implications for research and practice

Considering how little is known about reading interventions for older students with reading disability, future intervention studies should include pre- and post-outcomes of word reading skills and language. Future analysis should consider additional components of reading (i.e., vocabulary) to better understand the variance of performance and the characteristics of readers (Kulesz et al., 2016). For older students, the addition of specific language skills embedded within reading interventions and assessing the development of those skills may further contribute to understanding response to instruction (Foorman et al., 2020). Analysis of subgroups of students with most severely impacted performance in word reading and also of high school English learner participants would contribute to deepening the understanding of reader characteristics and the generalizability of the findings from this study.

One possible hypothesis to contextualize the findings of this study is through careful understanding of the intervention components. The word reading component in this study is a well-known intervention protocol (Archer et al., 2005). The word study component of REWARDS focuses on both syntactic and semantic parts of multisyllabic words. It may be that this specific type of instruction at the word level in addition to the vocabulary instruction provided is both supporting students’ ability to process connected text more with more accuracy resulting in increases in comprehension. This shift of instruction from phase 1 and phase 2 provides a plausible explanation for the findings from our second research question.

Other models of reading posit that accurate word reading alone does not necessarily lead to gains in comprehension because of the dependency on knowing the meanings of words (Perfetti, 2010). In a recent study of adolescent readers, vocabulary and background knowledge of readers were the best predictors of comprehension based on an explanatory item response analysis that examined how passage features, question types, and reader characteristics influence performance on reading comprehension (Kulesz et al., 2016).

For adolescents with different levels of word reading skills, practitioners should consider whether providing word reading interventions is appropriate and approach instructional decision-making based on initial word reading profile of students and from a position of flexibility dependent on the response to instruction. Students may benefit from continued instruction with word reading that is slightly reduced with more emphasis on other components in addition to comprehension such as reading fluency and vocabulary, specifically on building semantic and syntactic knowledge. High school students in the low average range such as the participants in this study may not need intensive word reading instruction. However, students with severely impacted word reading (i.e., more than 1.0 standard deviation below the norming average) may benefit from more time dedicated to building up word reading skills as ascribed by the decoding threshold hypothesis (Wang et al., 2019).
